# The Right Person, the Right Treatment, at the Right Time in Alzheimer's Disease: Insights From the 2025 Brain Aging Symposium

**DOI:** 10.1111/acel.70351

**Published:** 2025-12-29

**Authors:** Cecilia G. de Magalhães, Alibek Moldakozhayev, Maria Vina Lopez, Gene L. Bowman, Jasmeer P. Chhatwal, Manolis Kellis, Richard Mohs, Laura Nisenbaum, Yakeel T. Quiroz, Ravikiran M. Raju, Reisa A. Sperling, Mahdi Moqri, Vadim N. Gladyshev

**Affiliations:** ^1^ Division of Genetics, Department of Medicine Brigham and Women's Hospital, Harvard Medical School Boston Massachusetts USA; ^2^ Broad Institute of MIT and Harvard Cambridge Massachusetts USA; ^3^ Department of Neurology and Neurosurgery McGill University Montreal Quebec Canada; ^4^ Metabolic Disorders and Complications, and Brain Repair and Integrative Neuroscience Programs, Research Institute of the McGill University Health Centre Montreal Quebec Canada; ^5^ Department of Neurology Massachusetts General Hospital, NIA‐Massachusetts Alzheimer's Disease Research Center Boston Massachusetts USA; ^6^ Department of Neurology Massachusetts General Hospital, McCance Center for Brain Health, Clinical Trials Platform Boston Massachusetts USA; ^7^ Department of Neurology Massachusetts General Hospital, Harvard Medical School Boston Massachusetts USA; ^8^ Department of Neurology Center for Alzheimer Research and Treatment, Brigham and Women's Hospital, Harvard Medical School Boston Massachusetts USA; ^9^ Computer Science and Artificial Intelligence Laboratory Massachusetts Institute of Technology Cambridge Massachusetts USA; ^10^ Global Alzheimer's Platform Foundation Washington DC USA; ^11^ Alzheimer's Drug Discovery Foundation New York New York USA; ^12^ Department of Psychological and Brain Sciences Boston University Boston Massachusetts USA; ^13^ Department of Psychiatry Massachusetts General Hospital, Harvard Medical School Boston Massachusetts USA; ^14^ Division of Newborn Medicine Boston Children's Hospital, Harvard Medical School Boston Massachusetts USA; ^15^ Picower Institute of Learning and Memory Massachusetts Institute of Technology Cambridge Massachusetts USA; ^16^ Biology of Adversity Project Broad Institute of MIT and Harvard Cambridge Massachusetts USA

**Keywords:** Alzheimer's disease, biomarkers, brain aging, early diagnosis, precision medicine

## Abstract

On October 22nd, 2025, Brain Aging Symposium took place at Harvard Medical School bringing together leading researchers from academia and partner organizations to discuss recent advances in measuring and monitoring human brain aging trajectories, with a particular focus on Alzheimer's disease (AD). A central theme emerged: achieving “the right treatment for the right person and the right time” through precision medicine approaches. Key advances included the unprecedented validation of plasma‐based biomarkers, particularly brain‐derived p‐Tau217 that can identify seeding AD pathology with remarkable specificity, making large‐scale screening newly feasible. Integrating multi‐level “omic” modalities, spanning genetic information, molecular biomarkers of nutrition, lipid and protein signatures, neuroimaging measures, cognitive assessments, and lifestyle factors, enhances disease risk modeling and trajectory prediction beyond the capacity of any single marker. Early findings highlight critical roles for nutritional and lipid metabolism, and myelin integrity in brain aging, with cell and sex‐specific vulnerabilities identified in response to nutrition, social isolation, and metabolic stress. Computational approaches that combine single‐cell genomics, epigenomics, and artificial intelligence have been shown to accelerate causal discovery and therapeutic development. However, significant challenges remain: current biomarkers explain only half the variance in cognitive decline, racial and ethnic differences in biomarker levels lack mechanistic understanding, and scalable tools for comprehensive brain aging assessment are needed. The symposium underscored that preventing AD will require intervening during the preclinical asymptomatic phase. These multimodal screening platforms, coupled with mechanistically driven therapeutics, reduction in modifiable risk factors, including nutrition, vascular health, and social determinants of health, could profoundly impact the field.

## Graphical abstract

The Brain Aging Symposium highlighted how early detection and longitudinal tracking, enabled by multimodal biomarker integration, reveal critical roles for lipid metabolism, sex differences, and population heterogeneity in improving diagnostic accuracy and timing of intervention.

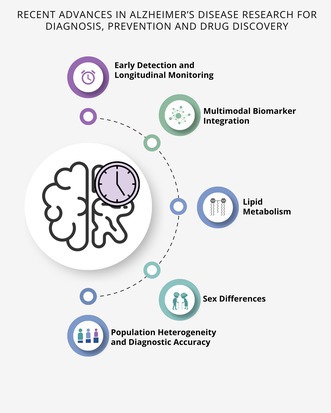

## 1 Introduction

**FIGURE 1:**
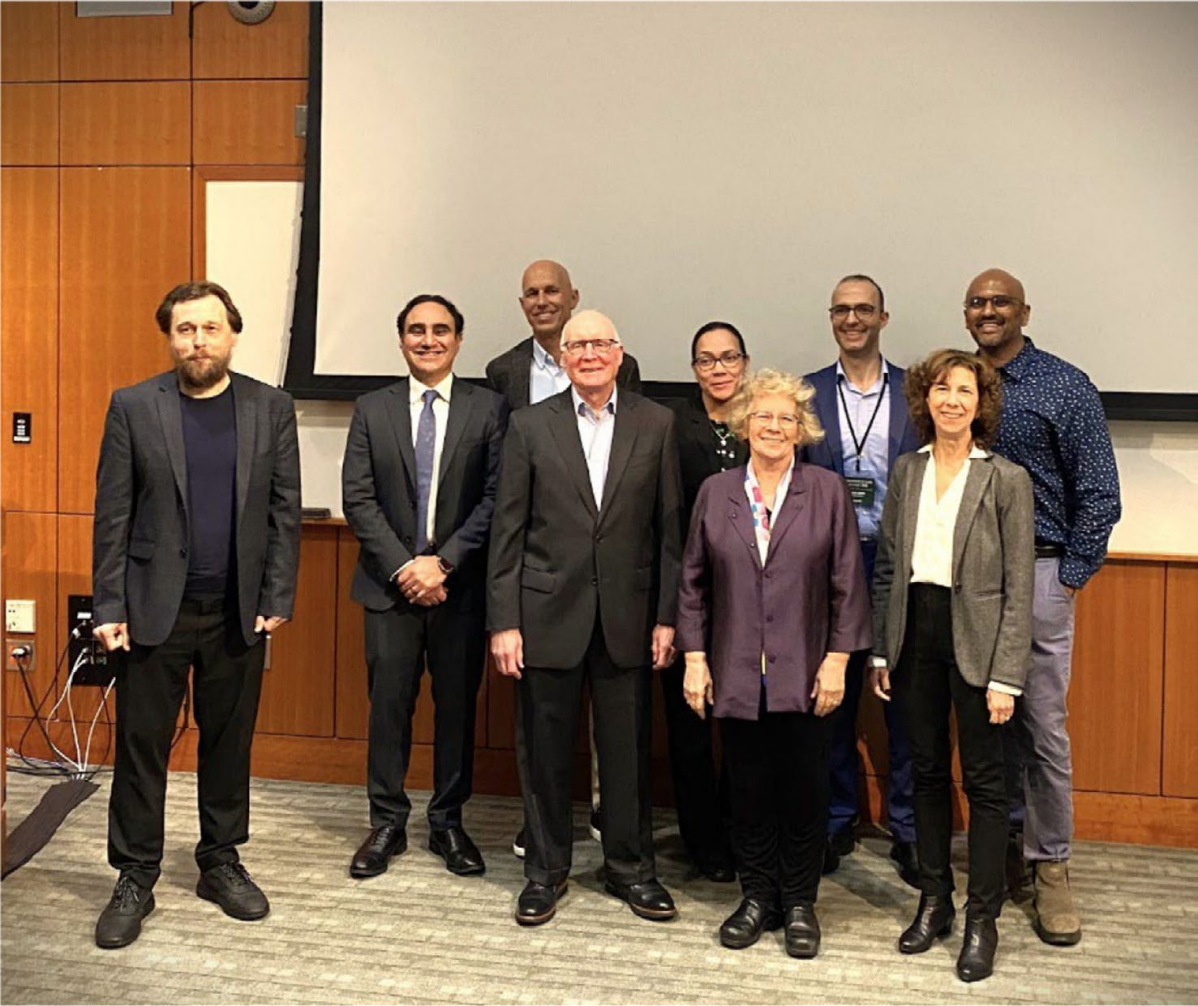
Brain aging symposium. Speakers and organizers of the Brain Aging Symposium held on October 22, 2025, at Harvard Medical School, Boston, MA. From left to right: Vadim Gladyshev, Jasmeer Chhatwal, Gene Bowman, Richard Mohs, Yakeel Quiroz, Reisa Sperling, Mahdi Moqri, Laura Nisenbaum, Ravikiran Raju.

The Brain Aging Symposium, organized by Biomarkers of Aging Consortium leaders—Vadim Gladyshev, Mahdi Moqri, Jesse Poganik, and Chiara Herzog—took place on October 22, 2025, at Harvard Medical School in Boston, MA. The event brought together leading researchers to discuss the latest advances in understanding and mitigating the aging process in the human brain, with a particular focus on delaying the seeding and progression of Alzheimer's disease (AD). The symposium featured eight cutting‐edge speakers representing both academia and partner organizations, including the Global Alzheimer's Platform and the Alzheimer's Drug Discovery Foundation (ADDF). Featured speakers included Laura Nisenbaum, Executive Director of Drug Development at the Alzheimer's Drug Discovery Foundation; Reisa Sperling Co‐Principal Investigator of the Harvard Aging Brain Study and Center for Alzheimer Research and Treatment; Gene Bowman, Clinical Trials and Brain Nutrition Lab Director at the McCance Center for Brain Health and Co‐PI of the PUFA Trial; Manolis Kellis, Head of the MIT Computational Biology Group; Yakeel T. Quiroz, Director of the Mass General Familial Dementia Neuroimaging Lab and the Multicultural Alzheimer's Prevention Program‐MAPP, and Jasmeer Chhatwal Co‐Investigators at the Harvard Aging Brain Study; Ravikiran Raju, Attending Physician at Boston Children's Hospital; and Richard Mohs, representing the Global Alzheimer's Platform (Figure 1). Together, the speakers provided a comprehensive overview of emerging strategies and collaborative efforts to better understand, monitor, and slow brain aging and neurodegenerative decline. Mahdi Moqri, Faculty member at Harvard Medical School, and Vadim Gladyshev, Professor of Medicine at BWH‐HMS, opened the symposium by highlighting the need to bridge aging biomarkers research with neurodegenerative disease studies, encouraging collaboration toward clinical translation. Further discussions highlighted the urgent need for both early detection and prevention of Alzheimer's disease, emphasizing emerging advances in body fluid‐based biomarkers, computational biology, nutrition, neuroimaging, and epigenomics as key areas driving progress in the field. In the broader perspective, the discussions converged on the importance of translating biomarkers into clinical trials and practice to guide patients toward the most effective therapeutic strategies. This vision reflects a recurring theme throughout the symposium: “the right person, the right treatment, at the right time”. This report summarizes the key presentations and discussions from the event, offering an integrated overview of the current landscape of brain aging research and its future directions.

## 2 Early Detection and Longitudinal Monitoring in Alzheimer's Prevention

Alzheimer's disease (AD), the most common form of dementia, took center stage throughout the symposium. A clear consensus emerged among the speakers on the critical importance of early detection and early intervention for effective intervention. The speakers emphasized that systematic, long‐term data collection is indispensable for biomarker discovery, particularly the establishment of robust baseline measurements capable of predicting individual disease trajectories. Several presentations showcased recent advances demonstrating how diverse methodologies can identify AD pathology decades before clinical symptoms appear. As noted by Dr. Laura Nisenbaum, Executive Director of Drug Development at the Alzheimer's Drug Discovery Foundation (ADDF), biomarker research has evolved from relying solely on invasive and costly modalities, such as cerebrospinal fluid (CSF) analysis and amyloid PET imaging, to developing accessible and scalable plasma‐based assays for detecting early pathological changes.

Dr. Reisa Sperling presented longitudinal findings from both the Harvard Aging Brain Study (HABS) and the Anti‐Amyloid Treatment of Asymptomatic Alzheimer's Disease (A4) Study, demonstrating the feasibility of clinical trials in preclinical AD populations (Sperling et al. 2023, 2024). The presentation highlighted the regulatory and conceptual challenges of conducting preclinical AD trials in individuals who show molecular signs of disease, such as amyloid accumulation, but remain cognitively asymptomatic. The A4 Study was the first large‐scale effort to test whether treating amyloid‐positive yet cognitively normal participants could delay or prevent cognitive decline. Launching this trial required close collaboration with the US Food and Drug Administration (FDA), marking a pivotal shift in how regulatory agencies approach prevention‐focused AD research. Dr. Sperling also discussed the ongoing AHEAD Study (AHEAD 3‐45), which builds on the foundation to determine the optimal stage for therapeutic intervention, extending even earlier into the preclinical phase of the disease (Rafii et al. 2022). A major innovation was moving plasma biomarker screening (using the C2N mass spectrometry assay) to the initial recruitment stage, which improved screening efficiency and enabled the study to screen over 20,000 individuals globally without excluding participants based on comorbidities or potentially biased cognitive tests. This approach revealed that even cognitively unimpaired individuals with elevated amyloid or p‐tau217 show reduced cognitive performance, reinforcing the rationale for intervening during the preclinical stage before irreversible cognitive impairment occurs.

Dr. Gene Bowman presented his group's work to develop and validate blood‐based measures of dietary quality and intake, and leveraging these “nutrient biomarkers” with data driven approaches to identify the nutrients and dietary patterns essential for neuroprotection. He began by presenting a pilot study evaluating the relationship between self‐reported food frequency questionnaire and objective plasma nutrient biomarkers, and how reliable these measures are among older adults with and without mild cognitive impairment evaluated at the NIA sponsored Oregon‐ADRC. The nutrient biomarker panel was reliable and was tested in association with total brain volume and white matter lesion volumes in older adults (Bowman et al. 2011, 2024). They identified distinct nutrient biomarker patterns associated with total brain volume and WML (Bowman et al. 2012). For example, the omega‐3 fatty acids, EPA + DHA explained 25% of the total variation in WML. Another vitamin profile explained significant variance in TBV. These data suggest that nutritional therapies should be targeted to specific features of brain health, a notion they have formally tested in a randomized clinical trial (PUFA Trial Shinto and Bowman, NIA R01), which examined the effects on WML progression and neuronal integrity breakdown in individuals aged 75 years and older harboring WML and suboptimal plasma omega‐3 levels at screening. The PUFA trial showed that omega‐3 slows neuronal integrity breakdown according to MRI DTI FA, and this effect is amplified in apoE4 carriers (Shinto et al. 2024). Dr. Bowman also placed nutrient biomarkers and their trajectory of change with aging in relation to cognitive decline and AD pathogenesis. For example, he presented ancillary study results in the Multidomain Alzheimer's Prevention Trial that a blood‐based nutritional risk index (NRI) explains both cognitive learning in the trial (from baseline) and incremental rates of cognitive decline over the three‐year trial with each point increase in NRI at baseline (Bowman et al. 2019). Then he presented the work of van Soest et al. (2024), showing that in people aged 50 and older with high NRI at a 4.7‐fold increase in dementia risk in the Framingham Study offspring cohort. These findings suggest that nutritional status is a reliable and consistent risk factor in mid and late life for dementia. Dr. Bowman also presented the MGH McCance Center for Brain Health Clinical Trials Platform, focused on early‐phase biomarker‐based clinical trials for prevention of AD pathology.

Dr. Manolis Kellis emphasized the critical importance of capturing the earliest molecular events in Alzheimer's disease. His presentation revealed that distinct brain cell types undergo profound yet highly specific transcriptional changes long before overt neurodegeneration occurs. These early alterations, particularly within vulnerable cell populations during the mild cognitive impairment (MCI) stage, may represent the most informative biomarkers for predicting disease onset (Blanchard et al. 2022; Mathys et al. 2024, 2019, 2023). These early molecular trajectories in vulnerable cell types, Dr. Kellis emphasized, represent critical targets for diagnosis and intervention before widespread pathological decline occurs.

Dr. Yakeel Quiroz presented findings from longitudinal tracking in familial AD cohorts, highlighting how pathology emerges far earlier than previously appreciated. In particular, the large Colombian PSEN1 E280A kindred offers a uniquely powerful longitudinal window on the preclinical stage because all carriers develop early‐onset AD. Using fMRI and structural neuroimaging, their group identified abnormal brain activation patterns and cortical alterations in APOE mutation carriers as early as childhood and adolescence (Fox‐Fuller et al. 2021; Quiroz et al. 2015, 2013). These results reinforce that AD‐related changes can precede cognitive symptoms by decades. Dr. Quiroz emphasized that such long‐term studies are crucial for identifying biomarkers capable of predicting individual disease trajectories and for distinguishing patients likely to experience rapid versus slow progression.

Similarly, Dr. Jasmeer Chhatwal presented evidence that neural alterations precede both cognitive decline and neurodegeneration. Drawing on data from the HABS, they examined longitudinal cognitive trajectories in asymptomatic individuals, revealing that many amyloid‐positive participants remain cognitively stable over time (Rabin et al. 2018, 2022; Yau et al. 2022). These findings highlight the need for additional biomarkers to identify those at greatest risk of decline. Several new blood‐based biomarkers—including phospho‐tau 217, neurofilament light chain, glial fibrillary acidic protein, and several other emerging markers of neuroinflammation—are well positioned to contribute to this goal and assist in the identification of older adults at imminent risk of AD‐related cognitive decline.

Further, Dr. Ravikiran Raju explored the mechanistic link between social experience and molecular aging, revealing how isolation accelerates the brain aging process. Their findings open new avenues for early detection and prevention, suggesting that understanding how social and environmental factors influence brain biology could help identify early biomarkers of vulnerability and guide interventions to enhance cognitive resilience before irreversible pathology occurs.

Finally, Dr. Richard Mohs emphasized that progress in early diagnosis hinges on large‐scale, coordinated biomarker validation efforts. Through the Global Alzheimer's Platform (GAP), their team launched the BioHermes studies to solve one of the field's central enigmas: the existence of individuals who are cognitively impaired but amyloid‐negative (Mohs et al. 2024). These studies also aim to evaluate alternative plasma‐based biomarkers, such as neurofilament light (NfL), that may more accurately capture early neurodegenerative changes in this population.

Together, these insights reinforce that early detection and precise diagnosis are essential for selecting the right individuals for intervention, and that longitudinal monitoring is critical for determining the optimal time to act. Prevention of Alzheimer's disease may therefore require intervening decades before the onset of cognitive symptoms, when the earliest molecular changes silently emerge.

## 3 Integration of Multiple Biomarkers Is Transforming AD Risk Prediction Accuracy

A consistent theme across the symposium was that no single biomarker can fully capture AD risk or trajectory. Integrating molecular, imaging, cognitive, and even digital measures provides a more comprehensive understanding of disease onset and progression. The next frontier lies in multimodal algorithms that combine these layers to predict individual risk, disease trajectory, and therapeutic response. However, beyond prediction, a fundamental challenge remains: distinguishing correlation from causation. Dr. Richard Mohs emphasized that large, integrative datasets combining multiple biomarker modalities are crucial for testing causal relationships and identifying which biological changes truly drive cognitive decline versus those that simply accompany it. Making this distinction is essential for advancing from biomarker discovery to effective treatment.

Dr. Yakeel Quiroz presented findings from her long‐term studies in the large Colombian kindred carrying familial AD mutations, demonstrating how the integration of genetic, plasma, and cognitive biomarkers reveals the earliest detectable disease phases. Her team emphasized the importance of establishing baseline levels of pTau217 and other biomarkers decades before symptom onset, while also uncovering resilience variants such as *APOE3 Christchurch*, which can delay cognitive decline despite amyloid accumulation (Quiroz et al. 2024). These findings shift the focus from identifying who will develop AD to understanding why some genetically at‐risk individuals remain resilient.

Dr. Jasmeer Chhatwal underscored that the amyloid burden alone provides an incomplete picture. Prediction models improve significantly when vascular risk, inflammation, and lifestyle factors are incorporated. He presented evidence that physical activity mitigates tau accumulation and preserves cognition even at modest levels, arguing that vascular, metabolic, and lifestyle factors should be viewed as integrated drivers instead of separate comorbidities in AD progression (Rabin et al. 2019).

Dr. Raju provided a powerful example of biological integration across scales. By combining behavioral, transcriptomic, lipidomic, and imaging data, his work linked social isolation to dysregulation of lipid metabolism and myelin integrity. Dr. Richard Mohs and Dr. Laura Nisenbaum highlighted the translation of multimodal biomarker discoveries into accessible diagnostic tools. Results from the BioHermes and BioHermes‐2 studies demonstrated that plasma biomarkers such as pTau217 and NfL can approximate the diagnostic accuracy of amyloid PET scans, potentially reducing the need for costly imaging. Together, these talks underscored a paradigm shift: from proof‐of‐concept imaging toward scalable, blood‐based, and multimodal platforms that can enable early detection, prevention, and precision intervention. Several speakers also highlighted pTau217 as the most robust plasma biomarker currently available, with eMTBR‐tau243 emerging as a complementary tool for tau staging and disease monitoring.

Recent advances in AI, single‐cell multiomics, and organoid systems are bridging this gap by uncovering mechanisms, identifying drug targets, and testing interventions at an unprecedented scale. These approaches are transforming how causality is inferred and therapeutics are developed. Dr. Manolis Kellis illustrated how large‐scale data integration and AI are driving precision medicine in neurodegeneration (Blanchard et al. 2022; Mathys et al. 2024, 2019, 2023; Xiong et al. 2023). His team's approach begins with genetics that provides causal insights, followed by single‐cell profiling of RNA and epigenomes in both healthy and diseased samples. By integrating these datasets, they identify driver genes, regulatory regions, and cell types, map the molecular circuitry connecting genetic variants to cellular dysfunction, and ultimately intervene in both cell cultures and mouse models. A key example of this pipeline: his team identified APOE4‐related cholesterol biosynthesis defects in oligodendrocytes. Even before symptoms appeared, APOE4 carriers showed reduced myelination due to impaired cholesterol transport in the endoplasmic reticulum. Restoring cholesterol transport using cyclodextrin improved myelination in human cell cultures and restored cognition in mice, directly linking genetic causality to therapeutic intervention (Blanchard et al. 2022).

Leveraging these molecular insights, Dr. Kellis's lab developed AI‐driven drug discovery pipelines that integrate protein structure modeling and chemical space exploration. Using generative AI models, his team clusters potential drug targets, predicts compound efficacy, and identifies novel or repurposed therapeutics. To experimentally test these predictions, they developed organoid‐based screening platforms using patient‐derived brain organoids that enable high‐throughput testing of hundreds of therapeutic candidates while capturing functional readouts. Together, these efforts define a next‐generation framework for mechanistic discovery and therapeutic translation: linking genetics to circuitry, circuitry to targets, and targets to AI‐designed therapeutics tested in patient‐derived models. This integrated approach accelerates causal understanding and enables precision treatment development tailored to individual molecular signatures in Alzheimer's and brain aging.

## 4 Lipid Metabolism and Myelin Integrity Are Emerging as Critical AD Pathways

Although amyloid beta and tau pathology account for a substantial portion of cognitive decline, they do not fully explain its course. Metabolic, vascular, inflammatory, and lipid‐related processes also contribute, and elucidating these pathways has become a central research priority that extends beyond amyloid and tau quantification.

Evidence presented at the symposium highlighted that social isolation and other environmental stressors can reshape brain biology, modulating lipid metabolism, myelination, and vulnerability to neurodegeneration, underscoring the need to integrate social determinants of health into biological models of brain aging. Dr. Raju showed that individuals reporting higher social isolation scores exhibit upregulation of cholesterol and lipid metabolism genes, while socially isolated male mice displayed decreased brain lipid content and thinner myelin sheaths. These findings reveal that disruption in lipid homeostasis and myelin biology may drive cognitive decline independent of amyloid and tau pathology, suggesting an evolutionarily conserved mechanism linking social isolation to myelin disruption and cognitive vulnerability.

Dr. Gene Bowman presented the PUFA trial showing that omega‐3 treatment can slow neuronal integrity breakdown among older adults, particularly apoE4 carriers (Shinto et al. 2024). Dr. Bowman shared results from in vitro studies in which omega‐3 were able to reduce the release of inflammatory cytokines from microglia. These findings suggest that nutritional biomarkers may help explain a significant portion of AD risk and highlight the need to integrate metabolic profiling into future predictive and preventive models. His research on nutrient biomarkers and metabolic profiles shows strong associations with brain structure, cognitive outcomes, and dementia risk. Optimizing nutritional status may represent an underexplored preventive strategy for brain aging.

## 5 Sex Differences in Vulnerability to Neurodegeneration

Dr. Manolis Kellis discussed published results where large‐scale single‐cell and multi‐regional transcriptomic analyses revealed profound sex‐ and cell‐type‐specific differences in the molecular landscape of AD and brain aging (Mathys et al. 2019). His group identified thousands of genes differentially expressed between males and females even before the clinical onset of AD, with male–female transcriptional differences nearly doubling with Alzheimer's Disease. Notably, white matter loss and myelination‐related pathways showed distinct regulation between sexes, where male individuals exhibited greater upregulation of genes linked to myelin synthesis, potentially reflecting a more effective compensatory response to white matter deterioration. Females, on the other hand, displayed a higher overall transcriptional burden, suggesting broader cellular stress responses (Mathys et al. 2019).

Dr. Raju presented compelling evidence that the effects of social isolation on brain aging are strongly sex specific. In both human and mouse data, males showed disrupted lipid metabolism, reduced myelin integrity, and elevated expression of cholesterol‐related genes, while females were largely protected from these molecular and structural changes. These findings suggest that male brains may be more vulnerable to the adverse neurobiological consequences of social isolation.

This sex‐specific vulnerability, observed in both humans and mice, underscores the importance of considering biological sex in studies when developing biomarkers of brain aging and therapeutics for AD.

## 6 Ensuring Diversity and Equity in Research Participation

Several talks emphasized the underrepresentation of diverse racial and ethnic groups in current cohorts, which may limit the generalizability of biomarker thresholds and predictive models. Expanding inclusive recruitment and culturally adapted methodologies is vital for equitable AD diagnosis and care.

Dr. Quiroz also emphasized the value and challenges of longitudinal cognitive testing in diverse populations. Her team adapted cognitive assessments to the Colombian cultural context, underscoring both the scientific importance of population‐specific tools and the ethical imperative of representation in Alzheimer's research (Fox‐Fuller et al. 2023; Giudicessi et al. 2024; Vila‐Castelar et al. 2020). These efforts exemplify how biomarker integration can improve prediction accuracy while ensuring that diagnostic and therapeutic advances are inclusive and globally relevant.

A key feature of the GAP‐led BioHermes initiative is its deliberate inclusion of participants from traditionally underrepresented populations, of whom nearly 30% of enrollees identified as Hispanic or African American. Dr. Mohs highlighted that while the base rate of amyloid positivity varies by ancestry, the sensitivity and specificity of plasma biomarkers remain comparable across racial groups. These findings reinforce the need to ensure diversity in biomarker validation cohorts to establish diagnostic tools that are applicable across populations.

Dr. Reisa Sperling also discussed emerging findings that highlight the importance of diversity in AD research. By moving plasma biomarker screening to the initial stage of recruitment, her team avoided excluding participants based on invasive or expensive tests and uncovered systematic differences in amyloid and tau pathology across racial and ethnic groups. Data from the AHEAD study showed that individuals identifying as Black, Asian, or Hispanic/Latino exhibit lower levels and prevalence of amyloid and tau biomarkers compared to White participants, even after accounting for age and *APOE* genotype. Dr. Sperling emphasized that the biological basis for these differences remains unclear but may involve distinct inflammatory or autophagic pathways that influence disease onset or progression. She underscored the urgent need for more representative cohorts and mechanistic studies to ensure that biomarker thresholds and diagnostic tools are equitable and applicable across diverse populations.

## 7 Future Directions in Alzheimer's Biomarker Discovery and Implementation

Despite major advances in biomarker discovery and validation, several challenges remain before we can achieve truly predictive and equitable approaches to AD and brain aging. The next phase of research must focus on combining multiple biomarkers, expanding our mechanistic understanding, improving diversity in study cohorts, and developing scalable tools for longitudinal monitoring of patients.

While plasma pTau and amyloid‐β markers explain approximately half of the variability in cognitive decline during aging, the remaining AD trajectory remains unaccounted for. As Dr. Reisa Sperling highlighted, identifying additional predictors, such as vascular, metabolic, inflammatory, or digital, is essential to capture the full spectrum of disease risk. Dr. Gene Bowman suggested that nutritional and metabolic factors may help explain a significant portion of cognitive decline and highlight the need to integrate those factors into future predictive and preventive models. Additionally, traditional cross‐sectional imaging and CSF measures capture only static snapshots of disease. In contrast, multi‐day data collection using wearable devices and digital cognitive tests can reveal dynamic changes in function and behavior. Integrating multiple biomarker modalities represents a promising path forward, allowing a more complete view of both vulnerability and resilience.

Multiple studies underscore that amyloid and tau levels differ significantly across racial and ethnic groups. These differences remain mechanistically unexplained but may involve variation in inflammatory or autophagic pathways influencing disease onset or progression. These gaps emphasize the need for low‐cost and scalable biomarker tools. Greater inclusivity will be key to building equitable and globally relevant brain health metrics.

Dr. Bowman and others emphasized the need to connect systemic metabolism to brain aging (Yassine et al. 2023). Current studies statistically adjust for BMI or adiposity, but rarely investigate the molecular roles of adipose and muscle tissue, or the signaling molecules they secrete. Understanding these metabolic interactions could clarify how lifestyle, diet, and genetics converge to influence AD risk and resilience, and may uncover modifiable targets for prevention.

Epigenetic profiling represents a promising next step for personalized prediction and therapeutic stratification. As illustrated by Dr. Manolis Kellis, global epigenome erosion was observed in late‐stage Alzheimer's patients, whereby regions of the genome that were previously accessible become repressed, previously inaccessible regions become active, and the boundaries between euchromatic and heterochromatin are blurred (Xiong et al. 2023). They also found global epigenomic differences between responders and non‐responders to immunotherapy revealed upstream regulators that guided effective combination treatments against cancer. Such approaches highlight how integrating epigenetic, transcriptomic, and proteomic layers can identify both therapeutic targets and patient‐specific responses.

Dr. Mohs and Dr. Nisenbaum discussed how the limited commercial incentives for diagnostics development in AD have historically slowed biomarker discovery compared to therapeutics. Dr. Nisenbaum noted that the recent FDA approval of the Fujirebio pTau217 assay marks a milestone in bringing biomarker science into clinical implementation. Through the ADDF's $100 million Diagnostic Accelerator initiative, her team aims to expand blood‐based biomarkers for population‐level screening and to guide personalized therapeutic strategies. Collaborative frameworks are transforming this landscape by pooling resources from foundations, pharma, and biotech to enable large‐scale studies. These partnerships exemplify how shared infrastructure can accelerate biomarker implementation, paving the way for broad clinical and public health use.

Finally, an overarching need identified across talks was the *creation of a standardized, scalable tool to quantify and track brain aging itself that could be used in clinics*. Such a measure could unify diverse data streams into a single, interpretable framework for both research and clinical monitoring. Developing such an index would mark a critical step toward precision prevention and personalized therapeutic guidance in neurodegenerative diseases.

## Author Contributions

C.G.M., A.M., M.V.L., M.M., and V.N.G. wrote the manuscript. All authors provided feedback and edits to their mentions.

## Funding

The authors have nothing to report.

## Conflicts of Interest

Y.T.Q. has served as a consultant for Biogen. R.A.S. has served as a consultant or on scientific advisory boards for AbbVie, AC Immune, Acumen, Alector, Apellis, Biohaven, Bristol Myers Squibb, Genentech, Ionis, Janssen, Novo Nordisk, Oligomerix, Prothena, Roche, and Vaxxinity over the past 3 years. R.A.S. has received research funding from Eisai and Eli Lilly for public‐private partnership clinical trials and receives research grant funding from the National Institute on Aging/National Institutes of Health, GHR Foundation, and the Alzheimer's Association. R.A.S.' spouse, K. Johnson, reports consulting fees from Bristol‐Myers Squibb, Janssen, Merck, and Novartis over the past 3 years. The other authors declare no conflicts of interest.

## Data Availability

The authors have nothing to report.
